# Codon usage patterns across seven Rosales species

**DOI:** 10.1186/s12870-022-03450-x

**Published:** 2022-02-05

**Authors:** Yao Zhang, Zenan Shen, Xiangrui Meng, Liman Zhang, Zhiguo Liu, Mengjun Liu, Fa Zhang, Jin Zhao

**Affiliations:** 1 College of Life Science, Hebei Agricultural University, Baoding, China; 2 Hebei Key Laboratory of Plant Physiology and Molecular Pathology, Hebei Agricultural University, Baoding, China; 3 High Performance Computer Research Center, Institute of Computing Technology, Chinese Academy of Sciences, Beijing, 100190 China; 4 Research Center of Chinese Jujube, Hebei Agricultural University, Baoding, China

**Keywords:** Rosales, Codon usage bias, Natural selection, Evolutionary relationships

## Abstract

**Background:**

Codon usage bias (CUB) analysis is an effective method for studying specificity, evolutionary relationships, and mRNA translation and discovering new genes among various species. In general, CUB analysis is mainly performed within one species or between closely related species and no such study has been applied among species with distant genetic relationships. Here, seven Rosales species with high economic value were selected to conduct CUB analysis.

**Results:**

The results showed that the average GC1, GC2 and GC3 contents were 51.08, 40.52 and 43.12%, respectively, indicating that the A/T content is more abundant and the Rosales species prefer A/T as the last codon. Neutrality plot and ENc plot analysis revealed that natural selection was the main factor leading to CUB during the evolution of Rosales species. All 7 Rosales species contained three high-frequency codons, AGA, GTT and TTG, encoding Arg, Val and Leu, respectively. The 7 Rosales species differed in high-frequency codon pairs and the distribution of GC3, though the usage patterns of closely related species were more consistent. The results of the biclustering heat map among 7 Rosales species and 20 other species were basically consistent with the results of genome data, suggesting that CUB analysis is an effective method for revealing evolutionary relationships among species at the family or order level. In addition, chlorophytes prefer using G/C as ending codon, while monocotyledonous and dicotyledonous plants prefer using A/T as ending codon.

**Conclusions:**

The CUB pattern among Rosales species was mainly affected by natural selection. This work is the first to highlight the CUB patterns and characteristics of Rosales species and provides a new perspective for studying genetic relationships across a wide range of species.

**Supplementary Information:**

The online version contains supplementary material available at 10.1186/s12870-022-03450-x.

## Background

Codons are the basic rules corresponding to the information carried by nucleic acids and proteins and are the basic link of information transmission in organisms. There are 61 types of codons encoding the 20 amino acids of natural proteins, and each amino acid has 1 to 6 synonymous codons that encode the same amino acid [1]. There is a widespread phenomenon of codon usage bias (CUB) in organisms, i.e., one species or gene usually tends to use one or several specific synonymous codons [2, 3]. The CUB pattern is affected not only by natural selection and mutation pressure [4] but also by the recombination rate [5], replication [6], GC content [4], gene length [7], hydropathicity, aromaticity and isoelectric point (pI) of the protein [8, 9], and protein secondary structures [10]. The use of codons is subject to selection pressure by these factors and is generally considered to be an important reason for the separation of species and the creation of new species [11, 12]. In some plants, the CUB patterns were also influenced by translational selection [13].

Different species not only have a preference when choosing different codons for the same amino acid, that is, they have different codon usage patterns [14], but they also have a preference when choosing a codon pair composed of two adjacent codons, and this preference is related to genome evolution. The mode of use of code pairs and the use of code pairs composed of adjacent codons in the genome have a preference, and the mode directly affects the efficiency of codon interpretation [15].

With the continuous improvement of genome sequencing technology, there have been an increasing number of studies on plant genome codons. However, most studies on plant codon preference have only been carried out in a single species. As there are few studies at the family or order level, we analyzed the CUB pattern of the plant genome at the order level. Rosales species such as apple, pear, peach, mulberry and jujube, have high nutritional and economic values. In this study, seven Rosales species with genome sequencing data were selected, and their GC content, neutrality plot, ENc plot, high-frequency codons and codon pairs, and GC3 variation were identified and compared systematically. Furthermore, a bi-clustering heat map among 27 plant species was established based on CUB patterns, and cluster analysis provided new insights for understanding their evolution.

## Results

### GC contents of 7 Rosales species

The GC contents of 7 Rosales species were compared and analyzed. As shown in Table 1, the AT content was high and the GC content was low in the 7 species, with an average GC content of 44.91%. Among them, the highest GC content was in *Morus notabilis* and the lowest was in *Ziziphus jujuba*.

**Table 1:** GC content of CDS across 7 Rosales species

Species	GC%	GC1%	GC2%	GC3%	GC3s%	The number of genes
*Ziziphus jujuba*	44.10	50.50	40.33	41.47	39.26	26,319
*Fragaria vesca*	44.91	50.99	40.29	43.45	41.26	30,405
*Malus domestica*	45.28	51.31	40.66	43.87	41.77	44,181
*Prunus mume*	44.57	51.01	40.44	42.24	40.06	22,850
*Prunus persica*	44.40	50.92	40.38	41.91	39.71	26,499
*Pyrus bretschneideri*	45.54	51.59	40.85	44.17	42.10	39,184
*Morus notabilis*	45.57	51.27	40.67	44.76	42.74	19,947
Average	44.91	51.08	40.52	43.12	40.99	

Note: GC1, GC2 and GC3 represent the GC content of the first, second, third base of codon; GC3s represents the GC content of the third synonymous position

Meanwhile, the average contents of GC1, GC2 and GC3 were 51.08, 40.52 and 43.12%, respectively, indicating that the species prefer A/T as the last codon. Previous studies have shown that dicot plants prefer to use codons ending in A/T and monocots prefer to use codons ending in G/C [16, 17]. In this study, the 7 Rosales species belong to dicot plants, and their use pattern in GC3 agreed with previous studies. Although the G/C content of codons in the 7 species was different (Table 1), their GC content distribution showed that the GC1 content was the highest, followed by GC3 and GC2, which is consistent with other plant species [16, 18].

The highest GC1 content was *Pyrus bretschneideri* and the lowest was *Z. jujuba*, the highest GC2 content was *P. bretschneideri* and the lowest was *Fragaria vesca*, and the highest GC3 content was *M. notabilis* and the lowest was *Z. jujuba* (Table 1). The GC3 and GC3s values of *Z. jujuba* were the lowest compared with those of the other species, indicating that its codons were not conserved and were more active during evolution [19].

### Neutrality plot analysis of 7 Rosales species

**Fig. 1:**
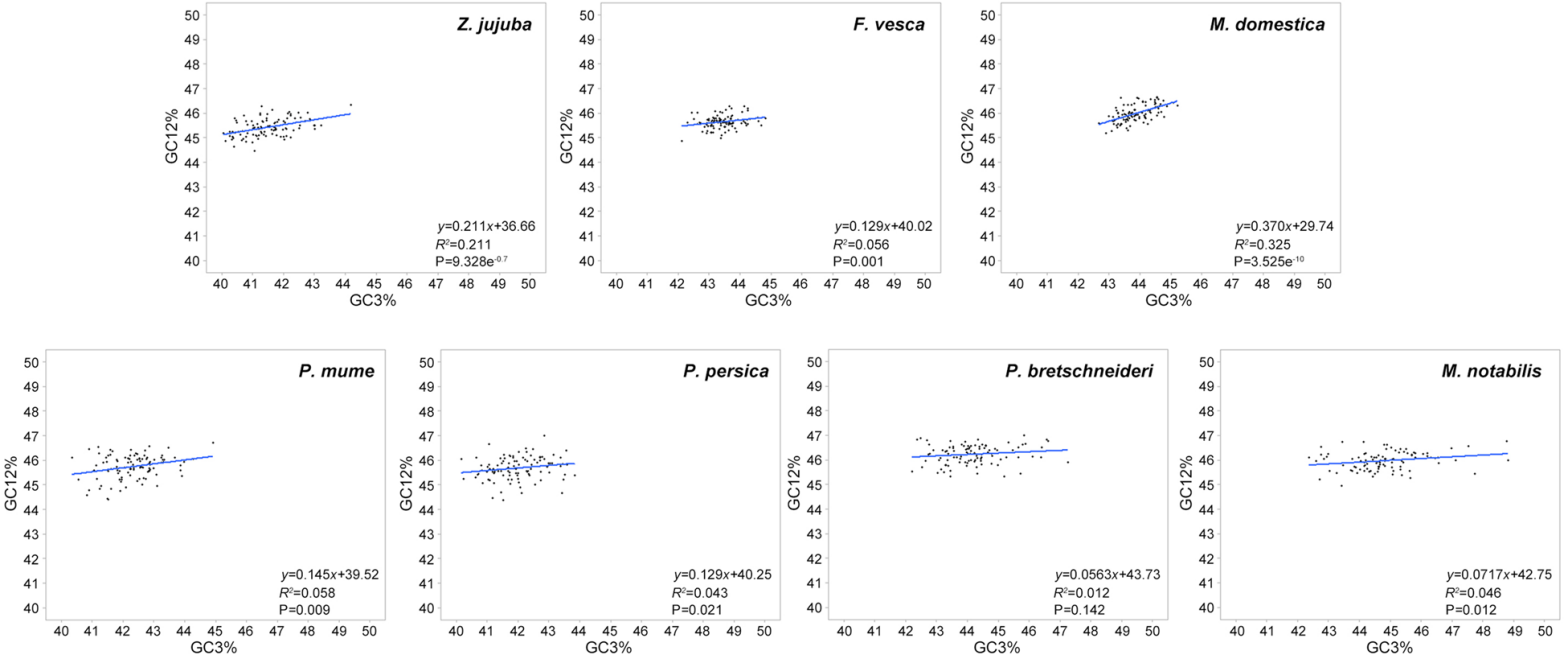
Neutrality plot of 7 Rosales species. The blue solid line represents the regression line. *P*-value is the correlation coefficient. If P-value was less than 0.05, and it showed that GC3 and GC12 was significantly correlated

Neutrality plot analysis was used to elucidate the correlation among the three codon positions, to identify the presence of selective mutation on CUB [20] and to quantify the extent of natural selection and mutation pressure. Through neutrality plot analysis (Fig. 1), it was observed that the GC12 (42.37% ~ 48.81%) and GC3 (39.22% ~ 47.26%) contents of the 7 Rosales species were distributed in a smaller range. A significant correlation were observed between the value of GC12 and GC3 in *Z. jujuba, F. vesca*, *M. domestica, P. mume, P. persica* and *M. notabilis* (*P* < 0.05). The slope of the regression line ranging from 0.056 to 0.370 among the 7 species, indicating that natural selection played a significant role in the CUB patterns. In addition, there were no significant correlations in *P. bretschneideri*, and its slope of regression line was near 0, indicating there was low mutation bias or high conservation of GC content [21]. The slope of the regression line of *M. domestica* was the highest (0.370), meaning that it was more affected by mutation pressure than the other Rosales species.

### ENc plot analysis of 7 Rosales species

**Fig. 2:**
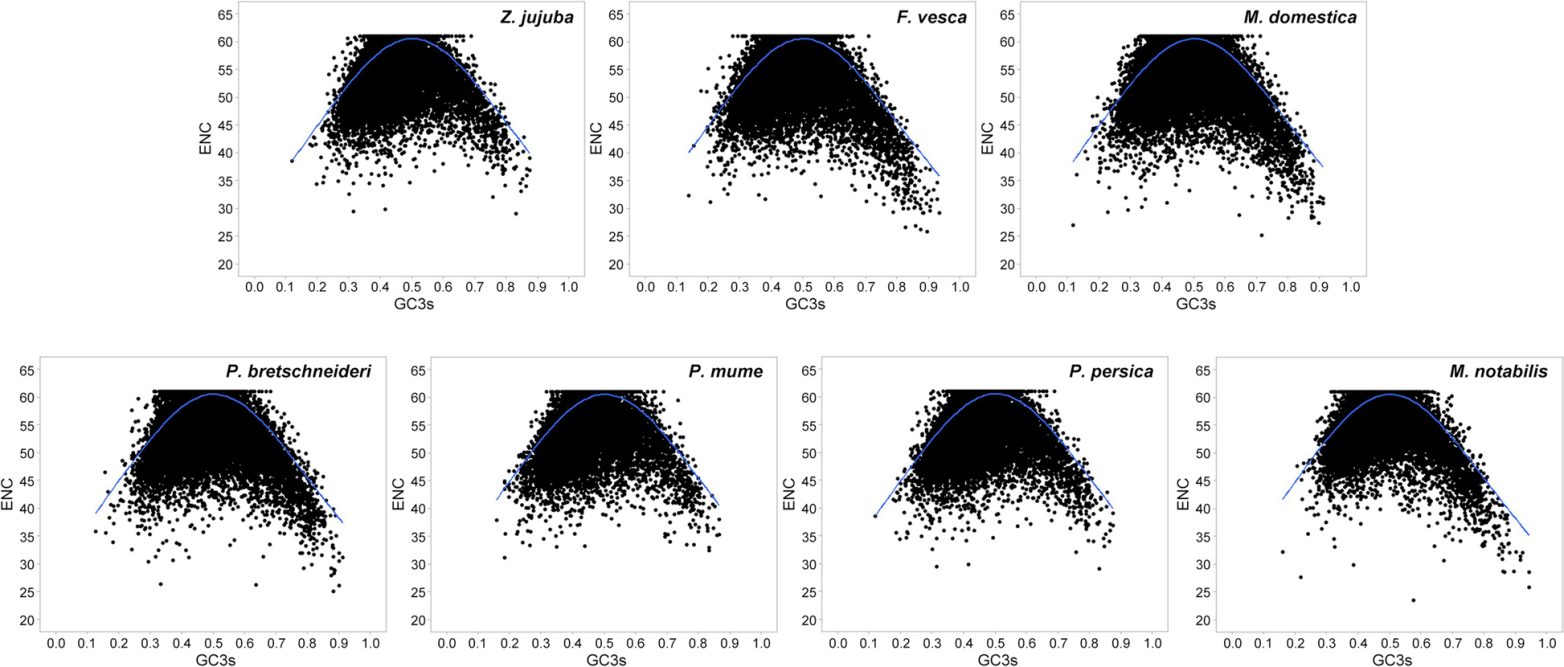
ENc plot of 7 Rosales species. The blue solid line represents the expected curve of positions of genes when the codon usage was only determined by the GC3s composition

Codon bias in a single gene is usually decided by the effective number of codons (ENC). To reveal the relationship between nucleotide composition and codon bias in the genome of the Rosales species, the ENC-GC3s map was analyzed (Fig. 2). The ENC values of the reconstructed genes ranged from 14 to 61, indicating that there were significant differences in codon bias among these genes [22]. Most genes are located below the expected ENc-plot curve, while only a small number of genes lay on or above the curve, indicating that mutation might be a weak factor shaping codon bias [23].

To obtain a more accurate estimate of the differences between observed and expected ENC values and to further prove the conservative influence of GC3s in Rosales species, the (ENCexp-ENCobs)/ENCexp was calculated. As shown in Table 2, the (ENCexp-ENCobs)/ ENCexp values of most genes ranged from −0.1 ~ 0.3, and more than 55% of the genes were distributed in the range of 0 ~ 0.1. The ENCs of 45% of the genes were different from the expected ENC values, indicating that mutation might be a weak factor in the evolutionary history of Rosales and the natural selection pressure might play a significant role in influencing the pattern of codon usage [24].

**Table 2:** Frequency distribution of (ENCexp-ENCobs)/ENCexp in 7 Rosales species (%)

Species	−0.2 ~ − 0.1	−0.1 ~ 0	0 ~ 0.1	0.1 ~ 0.2	0.2 ~ 0.3	0.3 ~ 0.4	0.4 ~ 0.5
*Z. jujuba*	0.21	7.22	57.59	30.28	4.17	0.48	0.05
*F. vesca*	0.07	6.07	61.55	28.49	3.36	0.44	0.02
*M. domestica*	0.07	8.05	66.55	22.33	2.67	0.30	0.02
*P. mume*	0.07	5.50	61.93	29.05	3.12	0.32	0.01
*P. persica*	0.07	4.91	63.59	28.30	2.81	0.30	0.02
*P. bretschneideri*	0.11	7.70	66.55	22.53	2.80	0.26	0.04
*M. notabilis*	0.09	7.68	67.20	22.79	2.00	0.23	0.02

### High-frequency codons and codon pairs in 7 Rosales species

The RSCUs of 64 codons were calculated, and the results showed that the number and content of high-frequency codons among the 7 species were different (Table 3) and that the AGA content was the highest. Six high-frequency codons were identified in *P. mume*, followed by five high-frequency codons in *Z. jujuba*, *F. vesca* and *P. persica*. Four high-frequency codons were identified in *M. domestica*, *P. bretschneideri* and *M. notabilis*. All 7 Rosales species contained three high-frequency codons, AGA, GTT and TTG, encoding Arg, Val and Leu, respectively.

**Table 3:** The top five high-frequency codons of 7 Rosales species

Species	Codon (RSCU)				
*Z. jujuba*	AGA(1.86)	GTT(1.58)	TTG(1.54)	TCT(1.51)	GCT(1.55)
*F. vesca*	AGA(1.86)	GTT(1.51)	TTG(1.52)	AGG(1.59)	GCT(1.56)
*M. domestica*	AGA(1.75)	GTT(1.55)	TTG(1.53)	AGG(1.58)	
*P. mume*	AGA(1.86)	GTT(1.57)	TCT/TTG(1.54)	AGG(1.60)	GCT(1.56)
*P. persica*	AGA(1.86)	GTT(1.58)	TTG(1.55)	AGG(1.58)	GCT(1.56)
*P. bretschneideri*	AGA(1.73)	GTT(1.55)	TTG(1.53)	AGG(1.58)	
*M. notabilis*	AGA(1.82)	GTT(1.54)	TTG(1.55)	AGG(1.52)	

**Fig. 3:**
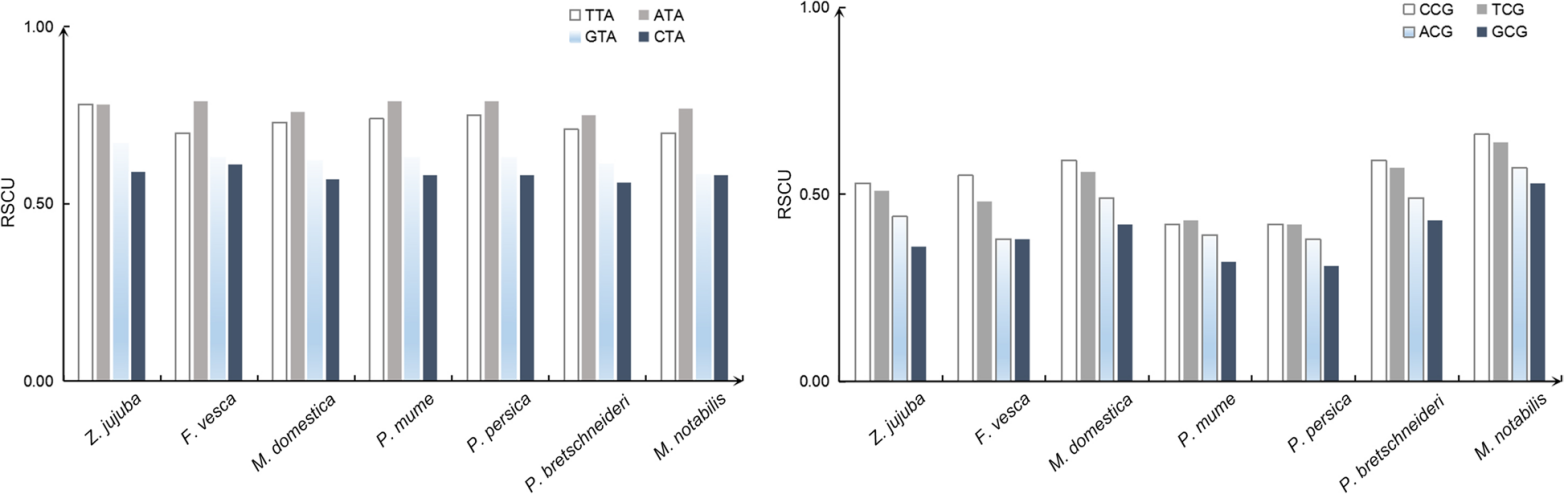
The RSCU value of NCG and NTA in 7 Rosales Species

It was observed that most of the identified high-frequency codons favoured codons ending in A/T. However, four NTA codons in 7 Rosales species had quite low RSCU values (Fig. 3), and the reduction in TA may increase protein production by inhibiting mRNA degradation [25, 26]. Four NCG codons also showed low RSCU values (Fig. 3), which may be conducive to avoiding possible mutations caused by DNA methylation [27, 28].

For stop codons, the average RSCUs of TGA, TAA, and TAG were 1.32, 0.89 and 0.77, respectively, and the results show that the 7 Rosales species prefer TGA as the stop codon (Additional file 1). The XCG/XCC ratio (Additional file 1) based on RSCU values was 0.73 (*M. notabilis*), 0.56 (*F. vesca*), 0.63 (*M. domestica*), 0.63 (*P. bretschneideri*), 0.57 (*Z. jujuba*), 0.49 (*P. mume*) and 0.49 (*P. persica*), indicating moderate CG dinucleotide suppression in the 7 Rosales species [29].

Overall, nnAAnn were the high-frequency codons and nnCCnn were the low-frequency codons among the 7 Rosales species (Table 4). In *F. vesca* and *M. domestica*, nnGCnn, nnCCnn and nnCTnn were the low-frequency codons, while nnCCnn and nnCTnn were the low-frequency codons in *P. mume* and *P. persica*, meaning their low degree of methylation at the genome level.

**Table 4:** Comparison of high-frequency codon pairs usage among 7 Rosales species

Codon Pairs	***Z. jujuba***	***F. vesca***	***M. domestica***	***P. mume***	***P. persica***	***P. bretschneideri***	***M. notabilis***
nnAAnn	9.38	11.07	12.54	11.56	12.53	12.51	12.21
nnACnn	3.72	4.49	4.79	6.41	5.17	4.30	4.32
nnAGnn	7.52	8.10	8.78	7.84	8.26	9.22	8.98
nnATnn	8.39	10.29	10.46	9.85	10.53	9.73	9.43
nnCAnn	4.45	4.57	4.92	5.42	5.09	4.98	4.59
nnCCnn	4.31	2.09	1.15	2.46	2.25	0.92	0.70
nnCGnn	1.90	3.23	1.29	0.32	0.15	1.33	2.38
nnCTnn	4.33	4.45	4.51	3.61	3.70	4.49	4.33
nnGAnn	9.16	10.04	11.64	9.65	9.10	11.01	10.63
nnGCnn	4.97	3.72	2.97	4.60	4.37	3.38	3.97
nnGGnn	5.38	6.59	5.33	6.12	5.49	6.07	5.40
nnGTnn	5.58	4.66	5.21	5.02	5.42	5.60	4.83
nnTAnn	7.63	7.17	5.89	6.01	6.09	5.38	7.29
nnTCnn	5.02	4.41	4.24	5.18	4.86	3.84	3.86
nnTGnn	8.62	6.71	8.06	6.84	6.94	8.16	8.07
nnTTnn	9.65	8.40	8.24	9.12	10.04	9.08	9.02

### GC3 content distribution

**Fig. 4:**
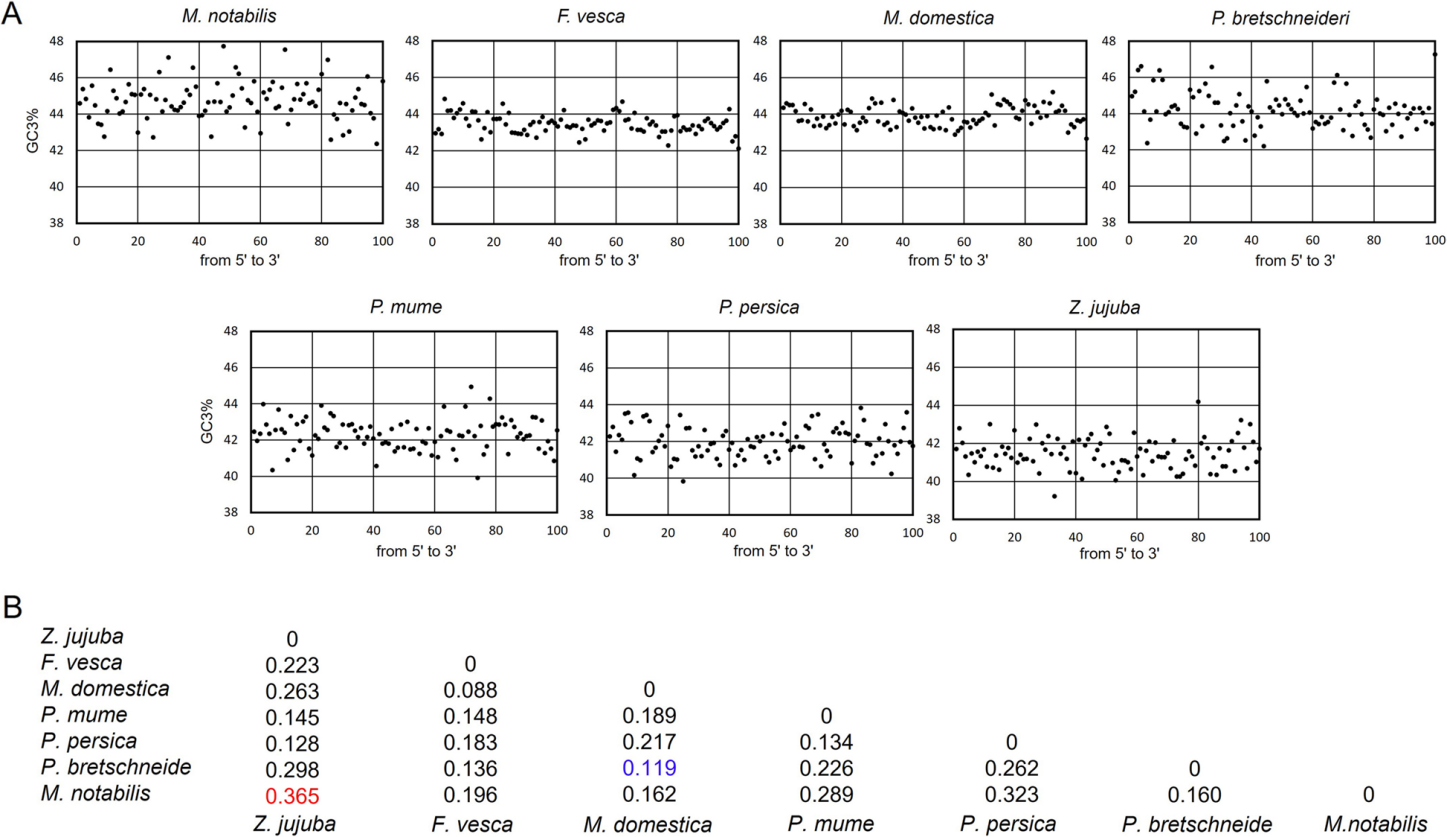
**A:** GC3 variation plot from 5′ to 3′ of 7 Rosales species. All genes in the species were divided into 100 groups equally, and each dot represents the average GC3 content of the genes in each group**; B:** The Euclidean distance of GC3 gradient between each of two Rosales species. The lower value of the Euclidean distance means the closer relationship. The maximum and the minimum values were marked in red and blue colors, respectively

To better explore the pressure on the 7 Rosales species during the evolutionary process, GC3 usage from 5′ to 3′ can reflect the transcription bias. Thus, we calculated and drew their GC3 content distribution map at the whole genome level. As shown in Fig. 4A, the GC3 contents of *M. notabilis*, *F. vesca*, *M. domestica* and *P. bretschneideri* were mainly distributed over 42%, while *P. mume*, *P. persica* and *Z. jujuba* were mainly distributed at approximately 42%. Among them, *Z. jujuba* had the lowest GC3 content distribution, which was more consistent with that of *P. mume* and *P. persica*; *M. notabilis* had the highest GC3 content distribution, which was similar to that of *P. bretschneideri*. The overall distribution of *F. vesca* and *M. domestica* was approximately 44%, and their distribution range was relatively concentrated.

To better understand the correlation of GC3 variations among the 7 Rosales species, their Euclidean distances were calculated (Fig. 4B), and ranged from 0.119 to 0.365. Among them, the Euclidean distance of *Z. jujuba* and *M. notabilis* was farthest, indicating that their relationship was farthest among the 7 species, while *M. domestica* and *P. bretschneideri*, with the closest Euclidean distance, were more closely related. The average Euclidean distance values of *F. vesca* and *M. domestica* were similar, and *Z. jujuba*, *P. mume* and *P. persica* also had a closer distance of the average Euclidean distance. The results further indicated that the same mutation pressure may contribute to the similar GC3 trends in the 7 Rosales species.

### Codon usage comparisons among 7 Rosales species and 20 other species

**Fig. 5:**
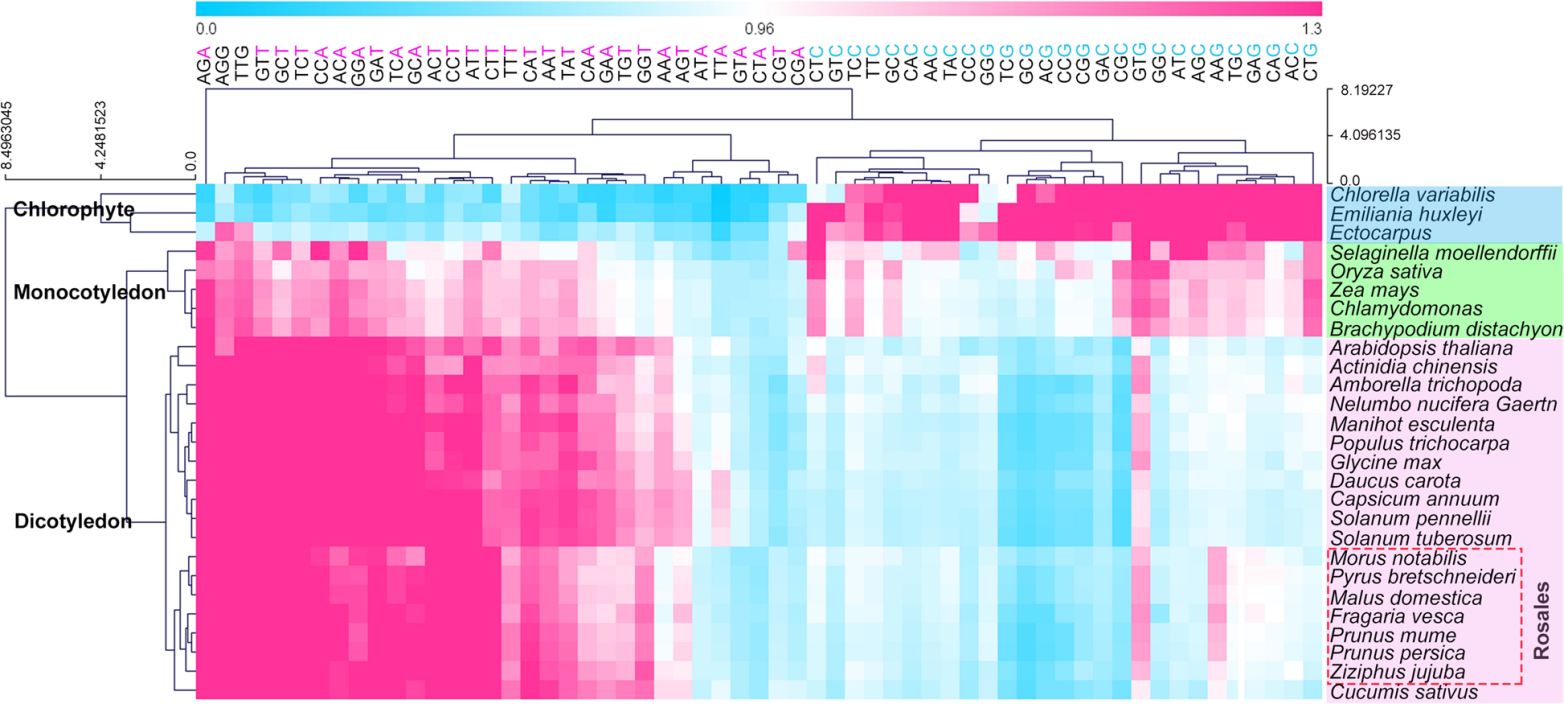
The bi-clustering heat map of RSCU based on 59 codons from 27 species using Euclidean distance and complete linkage clustering module. The blue, green and pink colors represent the Chlorophyte, Monocotyledon and Dicotyledon, respectively. The 7 Rosales species were marked in red box

To further explore the changes in CUB of Rosales species during plant evolution, we selected 20 other species for comparative analysis. To compare the RSCU values of all 59 synonymous codons (excluding Met, Trp, and three stop codons), a biclustering heat map was drawn to analyze the changes in CUB between these species during the evolution process. Based on the heat map (Fig. 5), the original chlorophytes and higher plants were clustered into two primary groups, and higher plants were divided into two branches, i.e., monocotyledonous and dicotyledonous plants. The evolutionary relationship of most plants was consistent with previous results based on genome data [30–32].

In the dicotyledonous subbranch, species from the same genus or closely related genera were clustered into small groups. Among them, the 7 Rosales species were clustered together, which was consistent with previous studies based on genome data [33, 34]. The results further suggest that codon preference analysis is also an effective method for analyzing the evolutionary relationships among various species.

Moreover, significant differences in codon preference between lower and higher plants were observed. Chlorophytes prefer using G/C as ending codon, while monocotyledonous and dicotyledonous plants prefer using A/T as ending codon (Fig. 5). For example, chlorophytes prefer using TTC encoding Phe, while monocotyledonous and dicotyledonous plants prefer using TTT. There were also different codon biases between monocotyledonous and dicotyledonous plants, such as they prefer to use AAG and AAA to encode Lys, respectively. From chlorophytes, monocotyledonous to dicotyledonous plants, the usage of A/T-ending codons increased gradually.

## Discussion

Genomic GC varies significantly among different species due to differences in mutational pressure [35]. In this study, the compositional properties and codon bias of the genes among 7 Rosales species were analyzed. The results of the composition analysis clearly revealed that the 7 species followed almost similar patterns of nucleotide composition, i.e., genes are A/T biased. The high tendency to use A/T over G/C mononucleotide-containing codons in the AT-rich dicot genome suggests that nucleotide composition, and not mutation bias, is an important factor of CUB. The AT-biased genomic architecture of coding sequences may be due to its relationship with evolutionary fitness.

To further explore the factors of codon bias during the evolution of Rosales, the results of the neutrality plot and ENC plot analysis found that natural selection was the main factor leading to codon bias during evolution and that mutation was a weaker influencing factor. In addition, translation efficiency and other factors also affect codon bias. This was consistent with previous reports on dicotyledonous plants, such as *A. thaliana* [16]. In contrast, studies in cyanobacterial genomes found that GC composition and environmental and mutation factors play important roles in influencing codon bias [36]. These results provide some clues for further research on the molecular evolution of the 7 Rosales species.

Through neutrality plot analysis, the correlation between the three codon positions can be clarified. In the 7 Rosales species, the correlation between GC12 and GC3 was significant, indicating that there were certain differences in the base composition of different codon positions, and there was a specific difference in the evolution mode of the third codon compared to the first and second codons [37]. If the correlation between the two positions was significant and the slope of the regression line is close to 0, then the codon preference was strongly affected by natural selection [38, 39]. The neutrality plot and ENc plot revealed that natural and human selection played a more important role than mutation pressure in CUB in the 7 Rosales species. The 7 Rosales species, except for *P. mume*, are very popular and important fruit trees, and they are strongly selected by artificial factors in cultivation. Thus, the results in this study also supported that the influence of natural selection on the evolution of Rosales is greater than that of mutation.

Nucleotide diversity is an important indicator to measure the level of genetic variation of species, and it plays an important role in studying the level of genetic polymorphism and genetic relationship of species. The codon preference of the plant genome can be analyzed and studied by a correlation index, and the frequency of codon usage between species at the order and family level is different; thus, the genetic relationship between species can be analyzed by a correlation index.

In the plant genome, the gene expression was directly related to the GC3 content, and the GC content was positively correlated with the gene length [40–43]. GC3s is also an effective method to study plant evolution, and the content of GC3s varies from Chlorophytes to Monocotyledon and Dicotyledon during plant evolution [43, 44]. This study showed that the GC1 and GC2 contents were relatively consistent from the mesh level analysis, indicating that they were very conservative indicators in species evolution, while the average GC3 and GC3s contents of genes were significantly different in different species (Table 1, Fig. 5). The content was similar among species within the same genera and was different among species from different families and genera, indicating that they were more affected by evolution factors.

The GC3 content varied across the 7 Rosales species’ transcripts, which could be dominated by strong mutational bias. GC3 usage from 5′ to 3’can reflect the transcription bias. CUB for a single type of codon is greatly influenced by the overall nucleotide content of the genome [45]. Extensive research on codon bias suggests that GC3 is the most important factor for genome evolution, and it also influences the gene expression level [17, 46]. Studies have shown that both the CUB patterns and the significant codon volatility values observed for *A. thaliana* are largely an effect of the GC content at the codon third position [40]. From the analysis of relevant indicators in 5 Rosaceae species, it can be seen that the frequency of use of high-frequency codons and special codons is relatively consistent among species within a family, while the frequency of use of high-frequency codon pairs is different (Table 4).

In previous studies, CUB analysis was mostly carried out in a single species or gene family within a species, while this study was rarely carried out in a range of species with large genetic differences at the family and order levels. This study provides a reference for the application of CUB analysis to elucidate genetic and evolutionary relationships among a wider scope of species and expands its application range.

## Conclusions

In this study, a series of CUB analyses showed that the 7 Rosales species were rich in AT and poor in GC. During the evolution of Rosales species, natural selection was the main factor leading to codon bias, and the influence of selection was greater than that of mutation. The use frequency of high-frequency codons and high-frequency codon pairs among the 7 species in the same family was relatively consistent, and there were obvious differences in the GC3 distribution between different families. This study is the first to highlight the CUB characteristics of Rosales species, which can help elucidate the mechanism underlying their molecular evolution and improve the expression levels of exogenous genes by codon optimization. It also provides a new perspective for studying genetic relationships across a wide range of species.

## Methods

### Sequence data collection and filtering

The dataset consists of two main parts. First, the protein-coding sequences (*.cds.fa.gz) of 7 Rosales species were downloaded from the NCBI database (https://www.ncbi.nlm.nih.gov/). Then, the genome and annotation data of 20 published plant species were downloaded (Additional file 2).

Protein-coding sequences (CDSs) of the compared plant species were extracted with Tbtools (http://cj-chen.github.io/tbtools/). The CDSs with no more than 300 bp, not having an ATG start codon, not ending with TAA, TAG or TGA stop codons, and having uncertain nucleotides and containing internal stop codons were filtered out by Python scripts written in-house. After filtering, the remaining high-quality sequences were used for further analysis.

### Indices of codon usage

The overall GC content and GC content at the first, second and third positions reflect the strength of directional mutation. RSCU (relative synonymous codon usage) is an index used to study the overall synonymous codon usage variation among genes. Codons with RSCU values over 1.0 were identified as positive CUB, and the values below 1.0 showed negative CUB. RSCU was calculated according to the formula described in Sharp and Li [47]. ENC (effective number of codons) reflects the degree of codon bias for 20 amino acids across ORFs. The ENC value is between 20 and 61. An ENC value close to 20 shows that only one of the synonymous codons is preferred, while a value close to 61 shows that each synonymous codon is used equally. GC content and RSCU analysis were performed with C++ programs written in-house, and ENC analysis was performed by the software codonW1.4.4 (http://codonw.sourceforge.net/).

### Analysis of GC content

GC content consists of the overall GC content, GC1 (GC content of 1st nucleotide in codon), GC2 (GC content of 2nd nucleotide in codon), GC3 (GC content of 3rd nucleotide in codon) and GC3s (GC content of 3rd synonymous codons).

### Neutrality plot and ENc plot analysis

A neutrality plot (GC12-GC3) was used to estimate and characterize the codon usage patterns among the three codon positions. GC12 represents the average of GC1 and GC2. A plot regression with a slope of 0 indicates no effect of directional mutation pressure (complete selective constraints), whiles a slope of 1 means the same mutation module between GC12 and GC3 and indicates that complete neutrality was the main factor in evolution.

The ENc plot (ENC-GC3s) is a general strategy to determine whether the codon usage of a gene is affected by mutation and selection. The expected ENc values were plotted against GC3s values and were calculated according to Eq. 1, where F represents the frequency of GC3s estimated [19]. The actual ENC values on or around the standard GC3s curve indicate that the codon bias is determined only by a G + C mutation bias. In other words, the values distributed far below the standard curve show that other factors, such as selection effects, are present [37].

$$\mathrm{ENc}=2+\mathrm{F}+\left(29/\left({\mathrm{F}}^2+{\left(1-\mathrm{F}\right)}^2\right)\right)$$

### Identification of high-frequency codons and codon pairs

Codons with an RSCU over 1.5 or having a relative frequency above 60% of the synonymous codons for the corresponding amino acids were identified as high-frequency codons. Codon pairs in which the last codon encodes the same amino acid were defined as synonymous codon pairs. High-frequency codon pairs were defined as codons with a relative synonymous codon pair usage (RSCPU) over 1.5 or codon pairs accounting for over 60% of the total number of synonymous codon pairs [44, 48]. Identification of high-frequency codons and codon pairs was performed by C++ programs written in-house.

### Comparison and cluster analysis

The RSCUs of 59 codons (excluding the 3 stop codons and codons with synonymous codons) from 7 Rosales species and 20 other plants were clustered by the Mev4.8.1 software (https://sourceforge.net/projects/mev-tm4/). Hierarchical clustering, Euclidean distance and sample tree parameters were set to cluster the RSCUs.

### Statistical analysis

CodonW1.4.4 software was used to analyze the indices of codon usage. The linear regression equation, R^2^ value and *P* value were calculated using MATLAB (version 7.0).

GC3 gradients from 5’ to 3’ reflect the variation trend of genomes. The calculation of Euclidean distance was performed by SPSS Statistics [49]. The lower the Euclidean distance is, the closer between two species is. The Euclidean distance of GC3 gradient between two species can be characterized by the vector (p, q) and it is computed according to the equation. The GC3 gradients plot was performed by Microsoft excel.

$$d\left(p,q\right)=d\left(q,p\right)=\sqrt{{\left({p}_1-{q}_1\right)}^2+{\left({p}_2-{q}_2\right)}^2+\dots +{\left({p}_n-{q}_n\right)}^2}$$

## Supplementary Information

**Additional file 1: Table S1.** The RSCU of codon among 7 Rosales species.

**Additional file 2: Table S2.** The data information of 27 plant species used in this study.

## Data Availability

All data and materials are presented in the main manuscript and additional supporting file.
