# Supplementary material for: Codon usage patterns across seven Rosales species
Source: BMC Plant Biol. 2022 Feb 5;22:65. doi: 10.1186/s12870-022-03450-x (PMC8817548; doi:10.1186/s12870-022-03450-x)
Supplement: Supplementary file 1 — Additional file 1: Table S1. The RSCU of codon among 7 Rosales species. [file 12870_2022_3450_MOESM1_ESM.docx]

| **Table S1** The RSCU of codon among 7 Rosales species | | | | | | | |
| --- | --- | --- | --- | --- | --- | --- | --- |
|  |  |  |  |  |  |  |  |
| Species | *F. vesca* | *M.domestica* | *P. bretschneideri* | *P. mume* | *P. persica* | *M. notabilis* | *Z. jujuba* |
| CCG | 0.55 | 0.59 | 0.59 | 0.42 | 0.42 | 0.66 | 0.53 |
| TCG | 0.48 | 0.56 | 0.57 | 0.43 | 0.42 | 0.64 | 0.51 |
| ACG | 0.38 | 0.49 | 0.49 | 0.39 | 0.38 | 0.57 | 0.44 |
| GCG | 0.38 | 0.42 | 0.43 | 0.32 | 0.31 | 0.53 | 0.36 |
| TTA | 0.70 | 0.73 | 0.71 | 0.74 | 0.75 | 0.70 | 0.78 |
| ATA | 0.79 | 0.76 | 0.75 | 0.79 | 0.79 | 0.77 | 0.78 |
| GTA | 0.63 | 0.62 | 0.61 | 0.63 | 0.63 | 0.58 | 0.67 |
| CTA | 0.61 | 0.57 | 0.56 | 0.58 | 0.58 | 0.58 | 0.59 |
| TGA | 1.33 | 1.33 | 1.33 | 1.33 | 1.35 | 1.35 | 1.24 |
| AAA | 0.88 | 0.89 | 0.90 | 0.87 | 0.86 | 0.87 | 1.02 |
| TAG | 0.79 | 0.78 | 0.77 | 0.80 | 0.79 | 0.77 | 0.73 |
| CCC | 0.64 | 0.69 | 0.69 | 0.67 | 0.67 | 0.68 | 0.68 |
| TCC | 0.87 | 0.90 | 0.90 | 0.83 | 0.82 | 0.90 | 0.90 |
| ACC | 0.89 | 0.90 | 0.92 | 0.89 | 0.87 | 0.87 | 0.87 |
| GCC | 0.78 | 0.79 | 0.79 | 0.78 | 0.77 | 0.86 | 0.86 |
