# Supplementary material for: Codon usage patterns across seven Rosales species
Source: BMC Plant Biol. 2022 Feb 5;22:65. doi: 10.1186/s12870-022-03450-x (PMC8817548; doi:10.1186/s12870-022-03450-x)
Supplement: Supplementary file 2 — Additional file 2: Table S2. The data information of 27 plant species used in this study. [file 12870_2022_3450_MOESM2_ESM.docx]

| **Table S2** The data information of 27 plant species used in this study. | | |
| --- | --- | --- |
| Species | NCBI accession | Sources of data |
| *Ziziphus jujuba* | GCA_000826755.1 | https://www.ncbi.nlm.nih.gov/genome/?term=Ziziphus+jujuba |
| *Fragaria vesca* | GCA_000184155.1 | https://www.ncbi.nlm.nih.gov/genome/?term=Fragaria+vesca |
| *Malus domestica* | GCA_002114115.1 | https://www.ncbi.nlm.nih.gov/genome/?term=Malus+domestica |
| *Prunus mume* | GCA_000346735.1 | https://www.ncbi.nlm.nih.gov/genome/?term=Prunus+mume |
| *Prunus persica* | GCA_000346465.2 | https://www.ncbi.nlm.nih.gov/genome/?term=Prunus+persica |
| *Pyrus bretschneideri* | GCA_000315295.1 | https://www.ncbi.nlm.nih.gov/genome/?term=Pyrus+bretschneideri |
| *Morus notabilis* | GCA_000414095.2 | https://www.ncbi.nlm.nih.gov/genome/?term=Morus+notabilis |
| *Chlorella variabilis* | GCA_000147415.1 | https://www.ncbi.nlm.nih.gov/genome/?term=Chlorella+variabilis |
| *Emiliania huxleyi* | GCA_000372725.1 | https://www.ncbi.nlm.nih.gov/genome/?term=Emiliania+huxleyi |
| *Ectocarpus* | GCA_004764655.1 | https://www.ncbi.nlm.nih.gov/genome/?term=Ectocarpus |
| *Selaginella moellendorffii* | GCA_000143415.2 | https://www.ncbi.nlm.nih.gov/genome/?term=Selaginella+moellendorffii |
| *Oryza sativa* | GCA_001433935.1 | https://www.ncbi.nlm.nih.gov/genome/?term=Oryza+sativaL |
| *Brachypodium distachyon* | GCA_000005505.4 | https://www.ncbi.nlm.nih.gov/genome/?term=Brachypodium+distachyon |
| *Chlamydomonas* | GCA_004335715.1 | https://www.ncbi.nlm.nih.gov/genome/?term=Chlamydomonas |
| *Zea mays* | GCA_902167145.1 | https://www.ncbi.nlm.nih.gov/genome/?term=Zea+mays |
| *Amborella trichopoda* | GCA_000471905.1 | https://www.ncbi.nlm.nih.gov/genome/?term=Amborella+trichopoda |
| *Nelumbo nucifera Gaertn* | GCA_000365185.2 | https://www.ncbi.nlm.nih.gov/genome/?term=Nelumbo+nucifera+Gaertn |
| *Daucus carota* | GCA_001625215.1 | https://www.ncbi.nlm.nih.gov/genome/?term=Daucus+carota |
| *Solanum pennellii* | GCA_001406875.2 | https://www.ncbi.nlm.nih.gov/genome/?term=Solanum+pennellii |
| *Solanum tuberosum* | GCA_000226075.1 | https://www.ncbi.nlm.nih.gov/genome/?term=Solanum+tuberosum |
| *Arabidopsis thaliana* | GCA_000001735.2 | https://www.ncbi.nlm.nih.gov/genome/?term=Arabidopsis+thaliana |
| *Manihot esculenta* | GCA_001659605.1 | https://www.ncbi.nlm.nih.gov/genome/?term=Manihot+esculenta |
| *Glycine max* | GCA_000004515.5 | https://www.ncbi.nlm.nih.gov/genome/?term=Glycine+max |
| *Populus trichocarpa* | GCA_000002775.3 | https://www.ncbi.nlm.nih.gov/genome/?term=Populus+trichocarpa |
| *Actinidia chinensis* | [GCA_009663005.1](https://www.ncbi.nlm.nih.gov/assembly/GCA_009663005.1#!/eukaryotes/16401/_blank) | https://www.ncbi.nlm.nih.gov/genome/?term=Actinidia+chinensis |
| *Cucumis sativus* | GCA_000004075.3 | https://www.ncbi.nlm.nih.gov/genome/?term=Cucumis+sativus |
| *Capsicum annuum* | [GCA_000710875.1](https://www.ncbi.nlm.nih.gov/assembly/GCA_000710875.1#!/eukaryotes/10896/_blank) | https://www.ncbi.nlm.nih.gov/genome/?term=Capsicum+annuum+ |
